# The prevalence and impact of depression in primary systemic vasculitis: a systematic review and meta-analysis

**DOI:** 10.1007/s00296-020-04611-7

**Published:** 2020-06-04

**Authors:** Bradley Pittam, Sonal Gupta, Ashar E. Ahmed, David M. Hughes, Sizheng Steven Zhao

**Affiliations:** 1 School of Medicine, University of Liverpool, Liverpool, UK; 2 Department of Rheumatology, Southport & Ormskirk Hospital, Southport, UK; 3 Department of Biostatistics, Institute of Translational Medicine, University of Liverpool, Liverpool, UK; 4 Department of Academic Rheumatology, Liverpool University Hospitals, Liverpool, L9 7AL UK; 5 Musculoskeletal Biology, Institute of Life Course and Medical Sciences, University of Liverpool, Liverpool, UK

**Keywords:** Primary systemic vasculitis, ANCA associated vasculitis, Giant cell arteritis, Depression, Fatigue

## Abstract

**Objective:**

To describe the prevalence of depression among patients with primary systemic vasculitides (PSV); compare prevalence according to vasculitis type and against controls; and examine the impact of depression on PSV outcomes.

**Methods:**

We searched Medline, PubMed, Scopus and Web of Science using a predefined protocol in accordance with PRISMA guidelines. We included all studies that reported the prevalence or impact of depression in PSV. We also included polymyalgia rheumatica (PMR) given its association with giant cell arteritis (GCA). Meta-analyses of prevalence estimates were performed using random-effects models and reported as percentages (95% confidence interval).

**Results:**

We reviewed a total of 15 studies that described the prevalence of depression, categorised into small (*n* = 10) and large vessel vasculitis (*n* = 7). Pooled prevalence estimate for depression in a small vessel (predominantly ANCA-associated) vasculitis was 28% (95% CI 20–38%) with significant heterogeneity (*I*^2^ = 93%). Depression prevalence in large-vessel vasculitis (Takayasu and GCA/PMR) was 24% (95% CI 17–34%), again with significant heterogeneity (*I*^2^ = 96%). One study reported 56% prevalence of depression in medium vessel disease. The prevalence of depression in small vessel vasculitis was higher than healthy controls. In these patients, depression and depressive symptoms were associated with poorer quality of life, adherence, and work disability, but not disease activity or damage.

**Conclusion:**

Depression is highly prevalent among patients with primary systemic vasculitis and associated with poorer outcomes across a range of measures in studies of small vessel disease. Further studies are needed for depression in medium and large vessel vasculitides.

**Electronic supplementary material:**

The online version of this article (10.1007/s00296-020-04611-7) contains supplementary material, which is available to authorized users.

## Introduction

Primary systemic vasculitides (PSV) are a group of rare, chronic diseases characterised by inflammation of the blood vessels [1]. They can affect vessels of various size and type, with potential for irreversible organ damage and death*.* Advances in treatment have improved both morbidity and mortality [2]. However, treatment regimens can be intense and are often accompanied by significant adverse effects. For example, glucocorticoids are known to increase the risk of a host of comorbidities from osteoporotic fractures to mood disturbances [3]. Long-term management can involve polypharmacy and a demanding schedule of follow-up with multiple specialties. The potential for unpredictable, organ or life-threatening relapses will also create significant psychological stresses. These factors can disrupt the life course with consequences on quality of life and mental health.

Priorities in follow-up clinics are often related to preserving life and organ function, reducing symptoms and managing physical comorbidities [4]. PSV research mostly reflects these clinical considerations, while mental health—a vital contributor to quality of life—can often be overlooked. Depression is more common in people with chronic disease than the general population [5]. It is associated with poorer outcomes in other rheumatic disease [6, 7]. Depression also has the potential to directly impact PSV management, for example, through effects on treatment adherence [8]. Improving our understanding of depression in the context of vasculitis management is, therefore, essential to facilitate patient engagement and partnership.

Despite the compelling reasons to study depression in vasculitis, research in this area are scarce and heterogenous. In this systematic review, our aims were to (1) describe the prevalence of depression among patients with PSV, (2) compare prevalence according to vasculitis type and against controls, and (3) examine the impact of depression on PSV outcomes.

## Methods

We performed a systematic review in accordance with the Preferred Reporting Items for Systematic Reviews and Meta-Analyses (PRISMA) guidelines [9]. We searched Medline, PubMed, Scopus and Web of Science for relevant literature in October 2019 using the following search terms: (vasculiti* OR *arteritis OR *angiitis OR ANCA OR (*neutrophil AND cytoplasmic) OR (*glomerular AND basement AND membrane) OR (Goodpasture* AND syndrome) OR (polymyalgia AND rheumatica)) AND (depress* OR (mental AND health) OR ((mental OR mood) AND (disorder* OR illness* OR dysfunction*))) NOT (case AND report).

We included all studies of primary systemic vasculitides (as specified in the 2012 Revised International Chapel Hill Consensus Conference [1]) that described clinically assessed or self-report depression. We additionally included polymyalgia rheumatica (PMR) because it is closely related to giant cell arteritis (GCA). Studies were excluded if they used non-representative sampling (highly selective recruitment or randomised controlled trials). Published conference abstracts were considered, as some prevalence studies may not be published as full articles but may have a sufficiently detailed methodology. Reviews, comments, and editorials were excluded.

Independent reviewers (BP, SG) screened titles and abstracts, assessed full-texts for eligibility and extracted data from qualifying studies. Any discrepancy at each stage was resolved through discussion moderated by a third reviewer (SSZ). Information from included studies was extracted into predefined tabulated summaries (Table 1), including vasculitis type and definition, sample size, country, age, gender, depression definition (including threshold used in screening questionnaires) and prevalence, and associations with vasculitis severity or disease activity. Studies were assessed for bias using adapted versions of the Newcastle Ottawa Scale (details in Online Supplementary Materials).

**Table 1:** Summary of 15 studies use for meta-analysis of depression prevalence

Study	Vasculitis	Definition	Sample size	Country	Mean age	%males	Depression definition	Prevalence
*Small vessel studies*								
Li 2018	GPA	Read code	570	UK	58.4	55.4	Read code	0.14
Hajj-Ali 2011	GPA	ACR criteria	55	USA	53	60	PHQ-9 > 9	0.22
McClean (abst) 2013	AAV	Unclear	151	UK	na	na	HADS > 10	0.27
Basu 2010	AAV	ACR criteria	66	UK	59	59	BDI > 12	0.15
Yun 2019	AAV	ACR criteria	61	South Korea	62.2	31.1	CESD-R > 15	0.46
Carpenter 2013	AAV	Self-reported	228	USA	51	30	CESD-R > 16	0.55
Koutantji 2003	AAV	ACR criteria	51	UK	63.06	59	HADS > 11	0.14
Grayson 2013	AAV	Self-reported	495	USA	na	33	Self-reported	0.53
Brezinova 2013	AAV	Physician diagnosis	93	Germany	55	46	BDI > 17	0.19
Hajj-Ali 2019	Cerebral angiitis	ICD	27	USA	50.8	51.9	PHQ-9 > 9	0.33
Grayson 2013	IgA vasculitis	Self-reported	12	USA	na	33	Self-reported	0.25
*Large vessel studies*								
Yilmaz 2013	Takayasu	ACR criteria	165	Turkey	41.3	7.3	HADS > 10	0.23
Alibaz-Oner 2013	Takayasu	ACR criteria	55	Turkey	42.3	10.9	HADS > 8	0.25
Grayson 2013	Takayasu	Self-reported	57	USA	na	6	Self-reported	0.43
Grayson 2013	GCA	Self-reported	32	USA	na	15	Self-reported	0.55
Li 2017	GCA	Read code	9778	UK	73.5	27.8	Read code	0.18
Vivekanantham 2018	PMR	Read code	550	UK	74.1	34	PHQ-8 > 9	0.15
Cawley 2018	PMR	Physician diagnosis	652	UK	72.6	38	PHQ-8 > 9	0.22
Brezinova 2013	LVV	Physician diagnosis	29	Germany	61	34	BDI > 17	0.1

Na, not available; GPA, granulomatosis with polyangiitis; AAV, ANCA-associated vasculitis; GCA, giant cell arteritis; PMR, polymyalgia rheumatica; LVV, large vessel vasculitis; ICD, international classification of diseases code; HADS, Hospital Anxiety and Depression Scale; CESD, Center for Epidemiologic Studies Depression Scale; PHQ, Patient Health Questionnaire; BDI, Becker’s Depression Inventory

We performed meta-analysis using random-effects models with logit-transformed prevalence estimates, using the inverse variance weighting method. Meta-analysis was performed separately for large and small vessel vasculitides. Prevalence estimates were then back-transformed and reported as percentages (95% confidence interval), additionally stratified by vasculitis definition. Heterogeneity of meta-analysis estimates was assessed using the *I*^2^ statistic. Funnel plots were used to assess the risk of publication bias. A range of screening tools was used for depression. To improve standardisation, we chose moderate depression where the prevalence of more than one severity was reported. This was approximated as Hospital Anxiety and Depression Scale (HADS) depression subscale > 10 [10]; Centre for Epidemiologic Studies Depression Scale Revised (CESD-R) ≥ 16 [11]; Patient Health Questionnaire (PHQ) > 10 [12]. We did not include the mental health component of short-form quality of life questionnaires (e.g., Short Form-36, SF-8) since there is evidence that they correlate poorly with depressive symptoms in vasculitides [13]. Analyses were performed using R version 3.6.2 and the “meta” and “metafor” packages.

## Results

The searched returned a total of 2508 publications. After deduplication, screening, and full-text assessment, 17 met eligibility criteria (15 for prevalence, and 2 additionally for the impact of depression). A flowchart of the selection process is shown in Supplementary Figure S1.

### Prevalence of depression in PSV

Details extracted from 15 studies that reported depression prevalence are shown in Table 1. Individual study sample sizes varied from 29 to 9978. Ten studies were of small vessel vasculitis; most of these were ANCA-associated vasculitis (AAV) [8, 13–20]. (Brezinova et al. reported results for small vessel vasculitis that were all AAV except one IgA vasculitis patient [18].) One study reported cerebral angiitis, which was included in the small vessel group [13]. One study reported results on medium vessel vasculitis (polyarteritis nodosa) [17]. Seven studies were of large vessel vasculitis, among which three were Takayasu arteritis and two GCA; we also included two PMR studies [14, 17, 18, 21–24]. (Brezinova et al. included polyarteritis nodosa in the large vessel group [18].)

Vasculitis was defined using ACR classification criteria in six studies [13, 15, 16, 19, 21, 22], diagnostic codes in five [14, 18, 23, 25, 26], self-report in two [8, 17], physician diagnosis in one [24] and unclear in one study [20].

Depression was defined by various means. Four studies used HADS [16, 20–22], 4 PHQ [13, 23, 24, 26], 2 Beck’s Depression Inventory (BDI) [18, 19] and 2 CESD [8, 15] were used with varying cut off values. One study used self-reported depression [17] and 2 diagnostic coding [14, 25].

The prevalence of depression in medium vessel vasculitis was available in only one study. Grayson et al. included 36 patients with polyarteritis nodosa, among whom 52% had self-reported depression [17].

### Meta-analysis for small vessel vasculitis

**Fig. 1:**
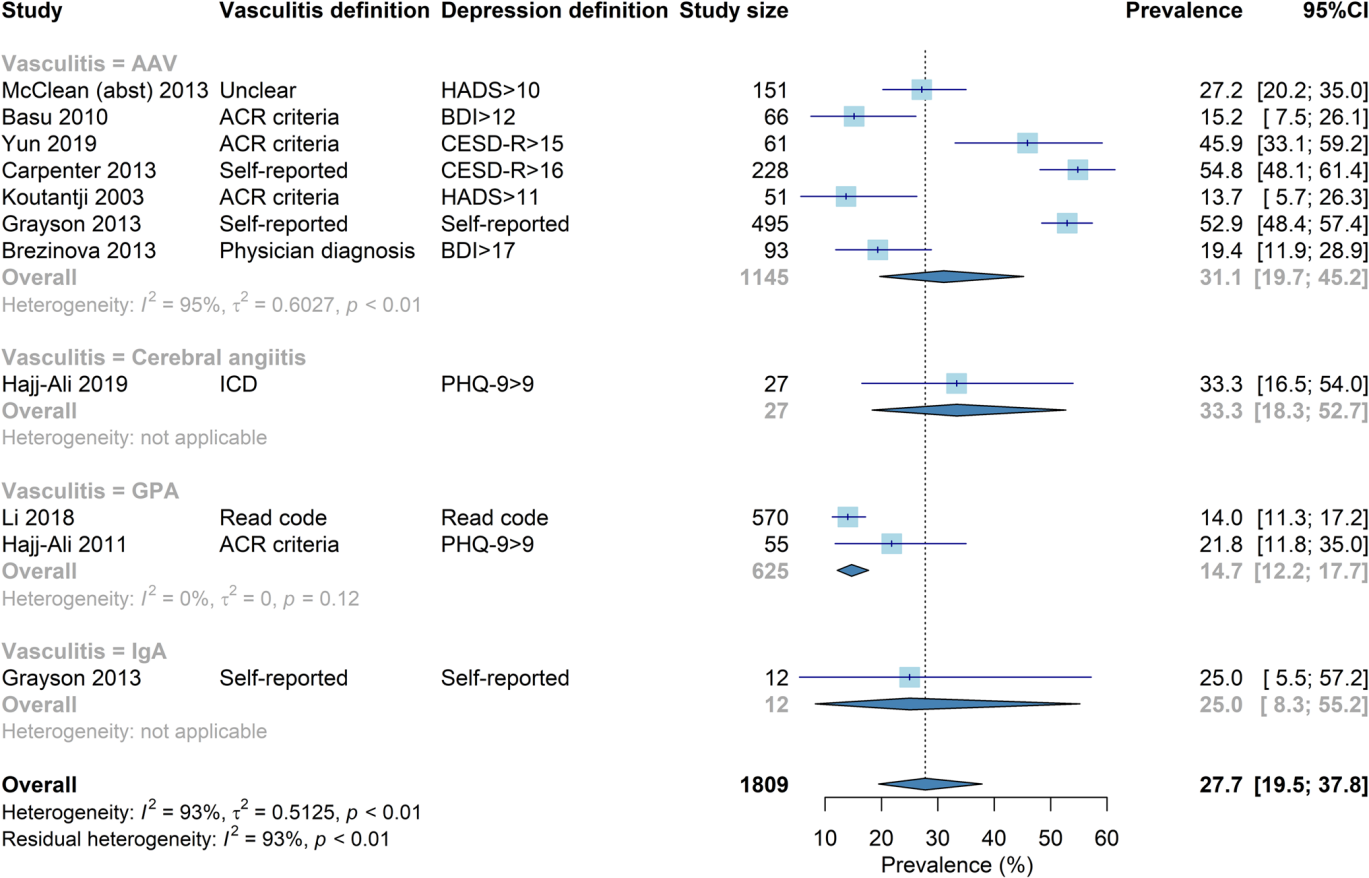
Forrest plot for the prevalence of depression in small vessel vasculitis, stratified by vasulitis definition. Corresponding funnel plot shown in supplementary materials

The pooled prevalence of depression in small vessel vasculitis was 28% (95% CI 20–38%). Prevalence ranged from 14 to 55%. There was significant heterogeneity (93%) that was not improved by stratifying them into subgroups (Fig. 1). Studies using self-reported depression and CESD-R generally reported higher prevalence.

### Meta-analysis for large vessel vasculitis

**Fig. 2:**
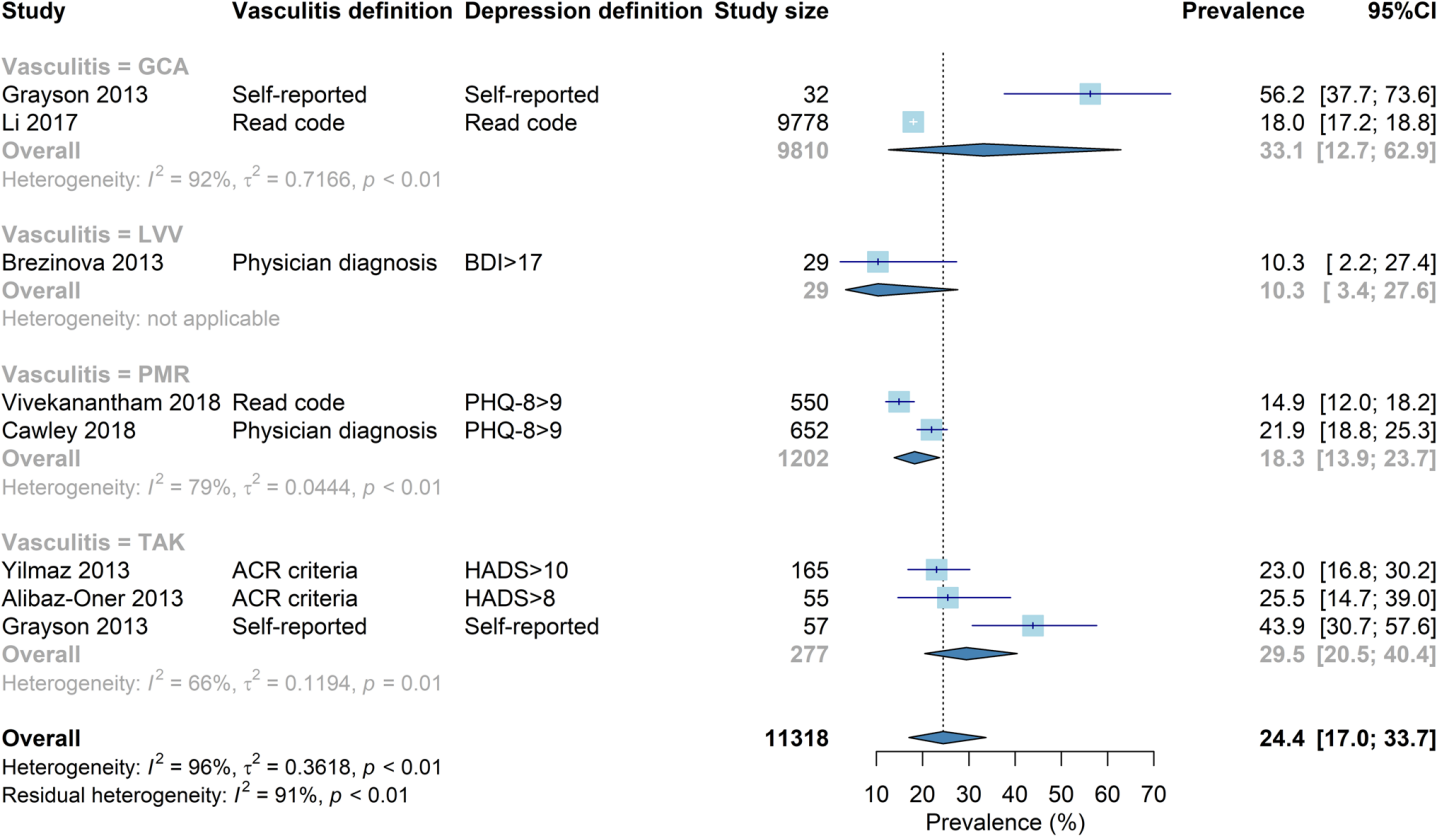
Forrest plot for the prevalence of depression in large vessel vasculitis, stratified by vasulitis definition. Corresponding funnel plot shown in supplementary materials

The pooled prevalence of depression in large vessel vasculitis was 24% (95% CI 17–34%). Prevalence ranged from 10 to 55%. Again, there was significant heterogeneity (96%) that was not improved by stratifying into subgroups (Fig. 2). Studies using self-reported depression generally reported higher prevalence.

### Depression compared between PSV and controls

Compared to non-vasculitis patients in UK primary care data, Li et al. reported an increased risk of depression among granulomatosis with polyangiitis (GPA) patients, although this was limited to the first 3 years of follow-up (HR 1.77) but not 3 years after diagnosis (HR 1.01) [14]. Hajj-Ali et al. also reported a higher prevalence of depression among GPA patients when compared to the general population (22 vs 7.6%; *p* < 0.001) [13].

Hinojosa-Azaola et al. reported a numerically higher prevalence of depression in AVV patients (9%) than RA (4%) and CKD (1%) controls, although this was not statistically significant (*p* = 0.29) [27]. Basu et al. found no significant difference in depression (BDI ≥ 13; AAV 15% vs primary care controls 21%; *p* = 0.50) [19].

For large vessel vasculitides, Alibaz-Oner et al. reported similar HADS depression scores between patients with Takayasu and healthy controls (5.1 vs 3.8; *p* = 0.168), although their sample size was small (*n* = 55 vs 40) [22].

### Associations between depression and disease outcomes

In a study of PSV (i.e., all vessel sizes), Brezinova et al. found BDI to be associated with poorer QoL (SF36 physical component score *b* = − 0.73, *p* < 0.001; mental component score *b* = − 0.56, *p* < 0.001) [18]. Koutantji et al. reported higher pain in PSV patients with depression (HADS depression scores in those with pain vs those with none/little: 7.6 vs 5.2, *p* < 0.01) [16]. Grayson et al. reported that a history of depression was associated with negative illness perceptions in PSV patients (OR 4.94; 95% CI 2.90–8.41); that is, organized beliefs that patients have about their illness that are important determinants of health-related behaviour [17]. Among patients with all types of vasculitis, Carpenter et al. showed depressive symptoms (CESD-R) to independently predicted non-adherence to medication (*b* = 0.01, *p* = 0.02) [8].

Among GPA patients, Hajj-Ali et al. did not find associations between PHQ-9 depression score and disease activity (Birmingham Vasculitis Activity Score; median 4 vs 3, *p* = 0.77), damage (Vasculitis Damage Index, median 0 vs 0, *p* = 0.23), or glucocorticoid requirement [13]. They did, however, report a significant association between PHQ-9 and fatigue (*r* = 0.73, *p* < 0.05). Hinojosa‑Azaola et al. also found an association between HADS and fatigue (*r* = 0.48, *p* = 0.01) among AAV patients [27]. When they dichotomised HADS scores (threshold > 7), those with depression or anxiety had more steroid use (prednisolone ≥ 10 mg/day; OR 6.65, 95% CI 1.37–32) than those without. These patients also had poorer quality of life (SF36 score 51.4 vs 66.8, *p* = 0.003) and more frequent sleep impairment (65 vs 33%, *p* = 0.01) than those without anxiety or depression. Similarly, Basu et al. found AVV patients with depression (HADS > 8) to have higher odds of having poor quality of life (OR 5.6; 95% CI 2.0–15.8) [28]. In a separate AAV study, Basu et al. also showed depression to be independently associated with unemployment (OR 4.4, 95% CI 1.8–10.8) [29].

There were no studies comparing depression and disease outcomes in large vessel vasculitides.

## Discussion

Depression was highly prevalent among patients with primary systemic vasculitides. Around 1 in 4 of those with either small or large vessel disease had depression, with no significant difference in meta-analysis estimates between the two groups. The prevalence of depression in small vessel vasculitis was higher than healthy controls. In these patients, depression and depressive symptoms were associated with poorer quality of life, illness perception, adherence, work disability, fatigue, pain, and sleep, but not disease activity or damage.

Pooled prevalence estimates for depression in PSV were higher than reported for rheumatoid arthritis (17% [30]), psoriatic arthritis (14% [31]) and ankylosing spondylitis (15% [6]). This may reflect the intensity of induction therapy or the severity of systemic inflammation, although most studies recruited stable patients in remission. It may also be explained by disruptions to the life course as a result of intensive follow-up, psychological stress from the multiorgan-threatening potential of unpredictable relapses, or higher cumulative doses of glucocorticoids. In systemic lupus erythematosus, where long-term glucocorticoids are also used, the prevalence of depression was reported to be 30–39% [32]. Glucocorticoids have well-known psychiatric side effects, but they also reduce inflammation which has been hypothesised to contribute to depressive symptoms [3, 33]. Hinojosa-Azaola et al. reported nearly sevenfold higher odds of requiring prednisolone ≥ 10 mg/day among those with depression [27]. This was replicated in the study by Koutantji et al. [16], but not Hajj-Ali et al. [13]. Patients with PSV are at risk of complications and comorbidities from the disease or its treatment. However, the only study of vasculitis disease activity and damage did not report an association with depression [13]. This may be because study samples were of patients with relatively low levels of disease activity and damage.

Depression was associated with poorer outcomes across a range of measures. Of most concern is the link between depression and poor adherence. This association is well documented in a variety of chronic diseases [34]. Symptoms of depression, including deficits in cognition, energy and motivation, and feelings of hopelessness, may contribute. Successful PSV management requires good adherence to maintenance therapy and engagement with healthcare services; therefore, it is imperative to address depression or depressive symptoms to optimise vasculitis management. Future studies should evaluate disease activity and other treatment outcomes in those with and without depression to quantify its impact. This may support the case for targeted screening and provision of mental health services. Some centres already offer parallel psychology services for vasculitis patients [35].

In patients with rheumatic conditions, depression remains underdiagnosed and under-treated [36]. This represents an area of unmet care need. Management of rheumatic diseases has evolved significantly over the last few decades, with the development of protocolised, target-driven disease management pathways. As a result, patient-encounters have become more pressured with less time to take an exhaustive history or perform a holistic assessment. Any assessment aids designed to promptly highlight the psychological aspects of rheumatic illness in a busy clinical environment should be considered for use. For example, it has been demonstrated that presenting patients with a checklist of potential concerns in the form of a ‘patient concerns inventory (PCI)’ prompted more discussion about various psychological aspects of illness, in comparison with the traditional consultation model [37]. While the PCI is a novel concept, there are several validated assessment tools to detect depression in a clinical setting including questionnaires cited in this review that could be incorporated into routine clinical practice.

A key strength of this review was the broad range of vasculitides considered. We were able to compare prevalence according to vessel size, as well as among subtypes. However, meta-analysis was limited by the small number of studies available and the variable definitions used for depression and vasculitis. Varying methods of assessing and defining depression could contribute to the high level of heterogeneity; for example, self-report may over-estimate prevalence, whereas diagnostic coding may under-estimate. Questionnaires also have differential sensitivity and specificity in detecting depression of various severity. The prevalence and impact of depression may be under-estimated among follow-up patients since those with severe depression are more likely to become lost to follow-up. Future studies would benefit from describing other related mental health diagnoses such as anxiety and suicidal intent. Depression is just one aspect of a well-recognised “symptom cluster”. These symptoms rarely occur in isolation and may share the same underlying mechanisms. Future studies should also address the impact of fatigue, sleep disturbance and fibromyalgia. As mortality outcomes continue to improve, quality of life becomes the most important outcome for these long-term conditions. Therefore, clinicians should actively seek and address these symptoms that are significant contributors to impaired health status.

In conclusion, depression is highly prevalent among patients with primary systemic vasculitis—higher than health controls and meta-analysis estimates from inflammatory arthritides. Pooled estimates were similar for small and large vessel disease. Depression was associated with poorer outcomes across a range of measures. However, these findings were mostly in small-vessel vasculitides and more studies are needed for medium and large vessel disease.

## Electronic supplementary material

Below is the link to the electronic supplementary material.

Supplementary file1 (DOCX 231 kb)
