# Supplementary material for: The prevalence and impact of depression in primary systemic vasculitis: a systematic review and meta-analysis
Source: Rheumatol Int. 2020 Jun 4;40(8):1215–21. doi: 10.1007/s00296-020-04611-7 (PMC7316669; doi:10.1007/s00296-020-04611-7)
Supplement: Supplementary file 1 — Supplementary file1 (DOCX 231 kb) [file 296_2020_4611_MOESM1_ESM.docx]

Supplementary materials for

**The prevalence and impact of depression in primary systemic vasculitis:** **a systematic review and meta-analysis**

Bradley Pittam, Sonal Gupta, Ashar E Ahmed, David M Hughes, Sizheng S Zhao

2,508 full-text articles generated through database search

711 duplicates removed

1,797 unique titles and abstracts screened

58 studies assessed for full-text eligibility

1,739 studies irrelevant or did not report prevalence or impact of depression

41 studies excluded

17 eligible studies (15 for prevalence, additional 2 for impact)

Supplementary Figure S1. Flowchart of study selection.

| Supplementary Table S1. Quality assessment using the Newcastle-Ottawa Scale | | | | | |
| --- | --- | --- | --- | --- | --- |
| Study | Representativeness | Sample Size | Vasculitis definition | Ascertainment of depression | Total |
| Alibaz-Oner 2013 | 0 | 0 | 2 | 2 | 4 |
| Basu 2010 | 2 | 0 | 2 | 2 | 6 |
| Brezinova 2013 | 1 | 0 | 1 | 2 | 4 |
| Carpenter 2013 | 1 | 0 | 0 | 2 | 3 |
| Cawley 2018 | 2 | 0 | 1 | 2 | 5 |
| Grayson 2013 | 2 | 0 | 0 | 0 | 2 |
| Hajj-Ali 2011 | 0 | 0 | 2 | 2 | 4 |
| Hajj-Ali 2019 | 0 | 0 | 1 | 2 | 3 |
| Koutantji 2003 | 1 | 0 | 2 | 2 | 5 |
| Li 2017 | 2 | 0 | 1 | 1 | 4 |
| Li 2018 | 2 | 0 | 1 | 1 | 4 |
| McClean (abst) 2013 | 0 | 0 | 0 | 2 | 2 |
| Vivekanantham 2018 | 2 | 0 | 1 | 2 | 5 |
| Yilmaz 2013 | 1 | 0 | 2 | 2 | 5 |
| Yun 2019 | 0 | 0 | 2 | 2 | 4 |
| Representativeness: 2 for population or primary care level data, 1 multi-centre, 0 single-centre or more selective. Sample size: 1 if justified, 0 if not. Vasculitis definition (“exposure”): 2 for classification criteria; 1 for physician diagnosis or diagnostic codes; 0 for self-report. Ascertainment of depression (“outcome”): 2 if validated tool or physician diagnosis; 1 if diagnostic code; 0 if self-reported. 0 is given if unclear or unreported. | | | | | |


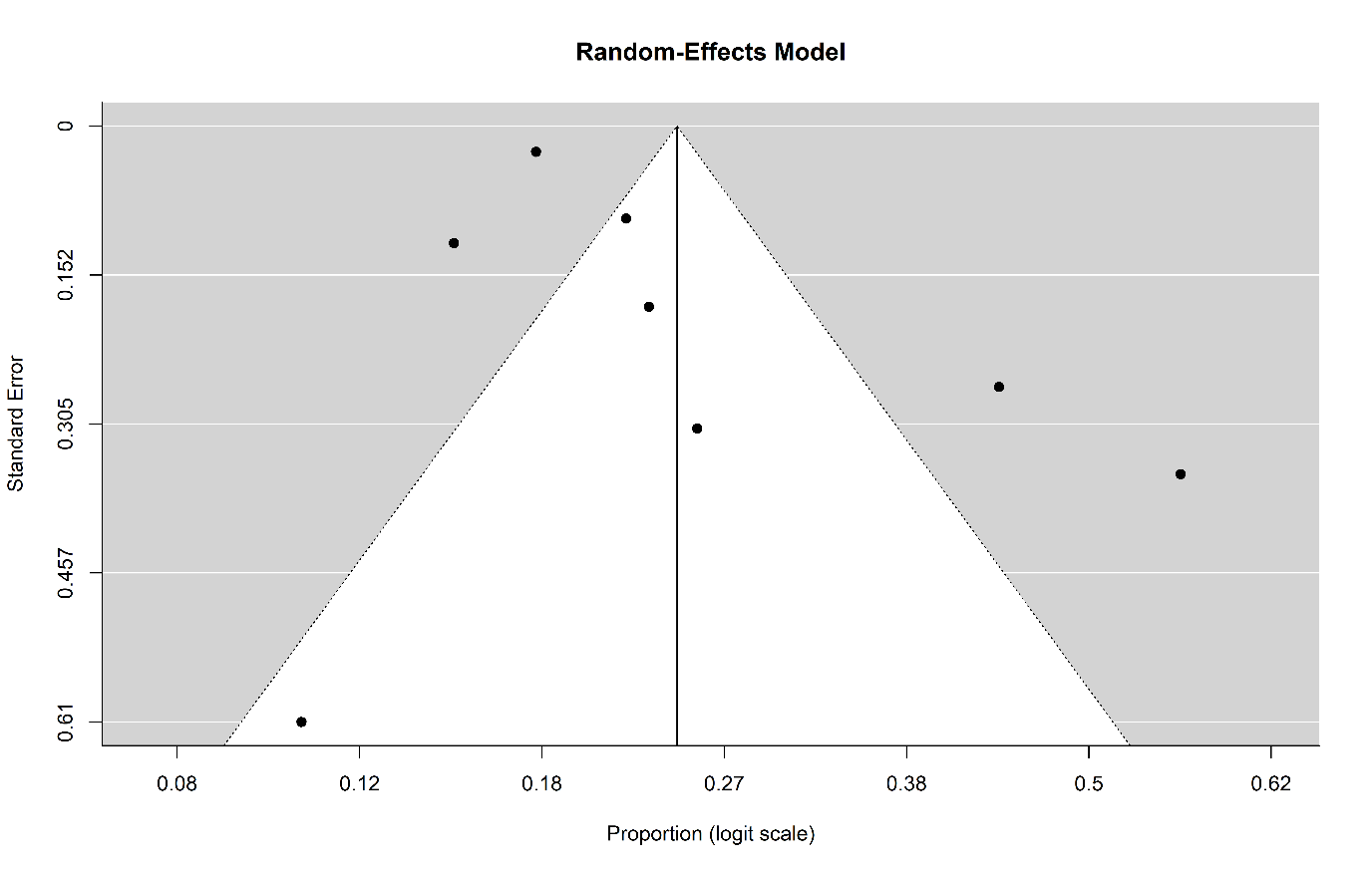


Supplementary figure S2. Funnel plot for small vessel vasculits meta-analysis.


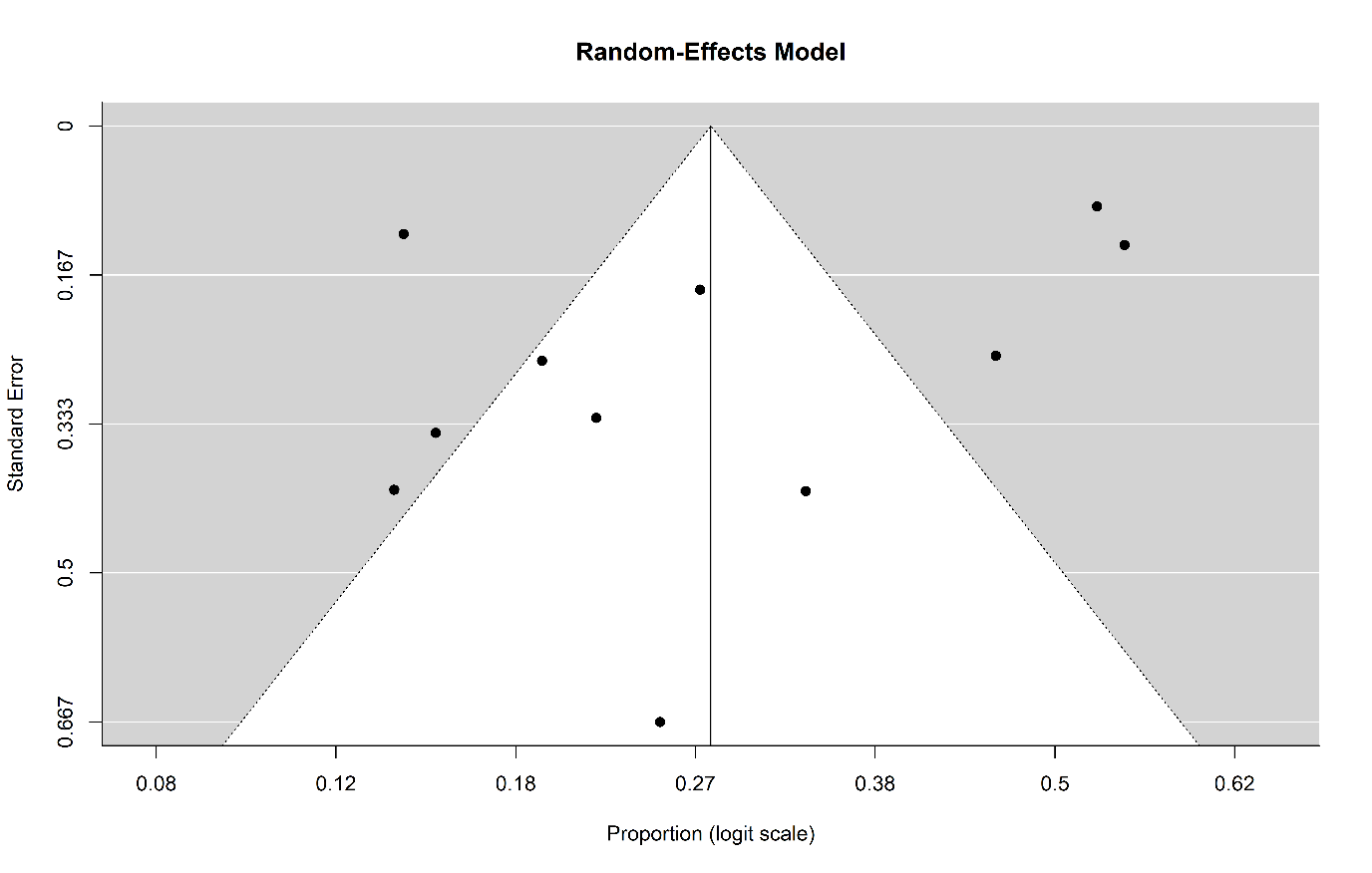


Supplementary figure S3. Funnel plot for large vessel vasculits meta-analysis.
